# Sleep, circadian rhythms and outcomes in intensive care unit

**DOI:** 10.3389/fneur.2026.1790266

**Published:** 2026-05-29

**Authors:** Marie-Anne Melone, Brian K. Gehlbach

**Affiliations:** 1 Service de Médecine Intensive-Réanimation, General Intensive Care Unit, Raymond Poincaré Hospital (APHP), Garches, France; 2 Departments of Internal Medicine and Neurology, University of Iowa, Iowa City, IA, United States

**Keywords:** critical illness, delirium, EEG, melatonin, post-intensive care syndrome, respiratory failure, weaning

## Abstract

Sleep and circadian rhythms are essential regulators of physiological homeostasis, influencing immune, metabolic, cardiovascular, and neurocognitive functions. In critically ill patients, these systems are frequently disrupted by the intensive care unit (ICU) environment, therapeutic interventions, and underlying illness. Increasing evidence suggests that sleep and circadian dyssrhythmias are associated with clinical outcomes during and after critical illness. This narrative review summarizes current knowledge on sleep and circadian rhythm alterations in adult ICU patients and examines their associations with short and long-term outcomes. We review studies using a wide range of assessment tools, including polysomnography, electroencephalography, actigraphy, cardiopulmonary coupling, hormonal profiling (melatonin and cortisol), temperature rhythms and clock gene expression. The relationship between these alterations and outcomes such as respiratory recovery, delirium, inflammation, illness severity, mortality, and post-intensive care syndrome is discussed. Across multiple studies, disrupted sleep architecture, characterized by reduced total sleep time, loss of rapid eye movement sleep, and atypical electroencephalographic patterns, has been associated with prolonged mechanical ventilation, weaning failure, and noninvasive ventilation failure. Alterations in sleep and circadian rhythmicity are also linked to delirium and markers of acute brain dysfunction. Circadian rhythm disruption, reflected by blunted or phase-shifted hormonal rhythms and altered clock gene expression, correlates with systemic inflammation, disease severity, and adverse prognosis. Emerging data further indicate that sleep and circadian disturbances may persist long after ICU discharge and contribute to cognitive impairment, psychological distress, fatigue, and reduced quality of life. Although methodological limits remain, the available evidence supports sleep and circadian rhythms as clinically relevant biomarkers of critical illness severity and brain dysfunction. Whether these represent modifiable therapeutic targets that can improve prognosis requires confirmation through rigorous interventional trials designed to account for the substantial confounding inherent in this population.

## 1 Introduction

Sleep and circadian rhythms are fundamental mechanisms to maintain homeostasis across a wide variety of physiological functions (1, 2). The sleep/wake cycle is the product of the homeostatic drive to sleep and the circadian timing system. The circadian timing system is a hierarchical system that consists of a central clock located in the suprachiasmatic nucleus (SCN) in the hypothalamus (3–5) and a widely distributed network of oscillators in peripheral tissues (i.e., brain, liver, heart, leukocytes, etc) (6). The SCN receives input via the retinohypothalamic tract from melanopsin-containing retinal ganglion cells in the eye and plays a key role in regulating melatonin secretion and sleep timing (7, 8). External cues, or zeitgebers, can entrain the SCN and other clocks throughout the body, with light being the most powerful cue (7).

Sleep that is restricted, disrupted, or mistimed can be both a sign of disease and a cause of poor health (9, 10). Light widely used throughout 24 h via indoor lights and electronic devices can alter clock gene expression and melatonin secretion with potential harmful effects on external and internal synchronization. For instance, jet lag and shift work are known to desynchronize internal oscillations and environmental cues leading to metabolic, cardiovascular, neoplastic, and mental health disorders (11, 12).

Disruptors of sleep and circadian rhythms are frequent in ICUs (13, 14), with objective polysomnographic studies demonstrating abnormal sleep architecture in 50–80% of critically ill patients (15, 16) and subjective sleep quality complaints reported by up to 60–70% of ICU survivors (17). Key factors include the 24-h provision of nursing care, weak day/night routines and light/dark cycles, prolonged bedrest, temperature cycles, feeding schedules, social interactions, administration of sedatives and other medications that affect the sleep–wake cycle (14, 18). Additionally, commonly administered medications including corticosteroids, norepinephrine, and beta-blockers may influence circadian biology (19), and strategic timing of these agents or clock gene modulation targeting BMAL1/CLOCK/PER/CRY could potentially serve as a circadian entrainment cue. Finally, pain, dyspnea and illnesses like sepsis influence sleep propensity and depth (16, 20).

Sleep and circadian disruption may make it more difficult for patients to recover from critical illness. For instance, a pilot study by Knauert et al. (21) found that many critically ill patients lose the normal diurnal variation in heart rate that can impact their cardiovascular adaptation to the disease. This study aimed to detect misalignment in circadian heart rate patterns and underscored how critical illness can uncouple physiology from the day-night cycle. In critical illness, sleep and circadian disruption may be simultaneously a consequence of disease severity, a useful biomarker of brain dysfunction, and a modifiable driver of impaired recovery.

Given the importance of sleep to health and the high prevalence of sleep and circadian dyssrhythmias in the ICU, the association between sleep and circadian disruption and clinical outcomes is deserving of careful study. Our review aims to summarize the literature on sleep and circadian dyssrhythmias and outcomes in ICU. We conducted a narrative review based on database PubMed until January 2026. Terms MeSH used were: (Sleep“[Mesh] OR “Circadian Rhythm”[Mesh] OR “Melatonin“[Mesh]) AND (“Critical Illness”[Mesh] OR “Intensive Care Units”[Mesh]) AND (“Ventilator Weaning”[Mesh] OR “Respiration, Artificial”[Mesh] OR “Delirium”[Mesh] OR “Rehabilitation“[Mesh] OR “Outcomes”). Inclusion criteria were: adults, hospitalized in intensive care unit, clinical studies, RCT, meta-analyses. We excluded studies with patient age under 18 years-old. Only articles in English were chosen.

We acknowledge that our search strategy specifically included “Melatonin” [Mesh] but not “Cortisol” [Mesh] as individual search terms. This decision reflected our focus on melatonin as the primary circadian phase marker in ICU research. However, studies examining cortisol rhythms were captured through the broader “Circadian Rhythm” [Mesh] term and through citation tracking of key articles.

## 2 Sleep and circadian rhythm analyses in the critically ill patient

Difficulties defining and evaluating sleep and circadian rhythms in critically ill patients have constrained progress in understanding how the circadian timing system interacts with critical illness and the ICU environment to modify clinical outcomes. Sleep has been assessed using validated and non-validated questionnaires, polysomnography (PSG), and actigraphy (22), while circadian rhythms have been evaluated through hormonal and clock gene measurements. PSG remains the gold standard, but the logistical and technical challenges involved in obtaining high-quality recordings in an artifact-rich environment currently limit its use. Moreover, the sleep electroencephalograph (EEG) of critically ill patients frequently lacks the typical features of sleep found in the general population. For instance, sleep spindles and K complexes are frequently absent, while the EEG in patients who are behaviorally awake may exhibit increased slow wave activity and decreased high-frequency activity similar to non-rapid eye movement (NREM) sleep (pathologic wakefulness) (23–26). Further complicating EEG interpretation is the frequent occurrence of abnormal EEG patterns in patients with a wide range of encephalopathies. These patterns include electrographic seizures with or without clinical manifestations and rhythmic or periodic EEG patterns consistent with the inter-ictal continuum, a spectrum of possibly harmful patterns that may increase neuronal metabolic demand and lead to secondary injury (27).

The widespread use of sedation in critically ill patients further complicates sleep assessment in this population. While normal sleep and alteration of consciousness from sedation may appear similar at the bedside and even on the EEG, normal sleep is cyclic, rapidly reversible, and temporally organized in ways the latter is not (26, 28). Sedation appears to possess some of the restorative properties of normal sleep (29), but is unlikely to provide the same complement of benefits, and its continuous administration is likely to have complex effects on sleep homeostasis and circadian rhythmicity. Sedation also has complex effects on the EEG, effects that vary according to sedative type and dose. For instance, low doses of propofol tend to induce paradoxical excitation with prominent beta-oscillations, which transitions to high-amplitude, slow-delta oscillations as the dose and depth of sedation increase (28). Benzodiazepines cause similar EEG changes. In contrast, the alpha-agonist dexmedetomidine has an EEG signature that more closely resembles NREM sleep, with preservation of sleep-like spindles at low doses (28) suggesting relative preservation of thalamocortical integrity when compared to other sedatives (30).

Specific staging rules have been suggested to score sleep in critically ill patients (23–25). In addition, novel EEG approaches have emerged as promising tools to characterize sleep/wake instability in critically ill patients. Metrics such as the Odds Ratio Product (ORP), spectral power analysis, and coherence measures allow objective quantification of cortical arousal (31). These methods provide insight into thalamocortical integrity and sleep depth beyond conventional staging (32). Limited-montage EEG, automated scoring using artificial intelligence, and emerging wearable EEG devices may enable standardized ICU sleep monitoring, though the findings generated by these devices will require careful interpretation and correlation with clinical findings before widespread implementation.

Various biomarkers of circadian dysrhythmias have also been analyzed in relation to ICU outcomes (see below). Interpretation of these studies is complicated by differences in the frequency of sampling, the type of biological sample used (urinary, salivary, blood), and the potential impact of diseases and treatments on these markers. For instance, urinary melatonin sampling has been used and shown interesting results that confirmed circadian rhythms alterations in the ICU (26). A few authors have been able to show a blunted rhythms and/or phase-delay of melatonin secretion in critically ill patients (26, 33–35), abnormalities that may be treatable with appropriately timed light therapy (34).

Neuroimaging modalities offer complementary approaches to assess sleep, though their application in ICU populations remains limited. Functional magnetic resonance imaging (fMRI) and positron emission tomography (PET) studies in healthy volunteers subjected to sleep deprivation have demonstrated altered cerebral blood flow and metabolic activity in key regions involved in attention and arousal (36, 37). However, the feasibility of advanced imaging in critically ill patients is constrained by transport, scan duration, sedation, and underlying brain pathology.

## 3 Short-term outcomes

### 3.1 Respiratory outcomes

Multiple studies suggest that poor sleep quality and disrupted sleep EEG patterns in ICU patients are correlated to respiratory recovery. In acute hypercapnic respiratory failure, Roche-Campo et al. (38) found that patients who eventually failed noninvasive ventilation had significantly worse sleep. These patients exhibited an atypical EEG pattern, severe circadian sleep-cycle disruption, and markedly reduced rapid eye movement (REM) sleep time. These patients were more likely to require intubation or prolonged ventilatory support. Delirium occurred in 64% of patient with NIV failure compared to 0% of those who succeeded with noninvasive ventilation.

Similarly, Thille et al. (39) reported that nearly half of difficult-to-wean ICU patients (failure after at least one spontaneous breathing trial) exhibited atypical sleep patterns. These patients took much longer to wean off mechanical ventilation (median 5 vs. 2 days) and often lacked any REM sleep. In multivariate analysis, the presence of atypical sleep was independently associated with prolonged (> 48 h after PSG) weaning, suggesting that acute brain dysfunction reflected in the sleep EEG can directly hinder breathing trials. One potential mechanism for a causal role of sleep deprivation is a reduction in cortical compensation for a respiratory load (40).

Recent prospective data reinforce that how patients sleep at the time of liberation from ventilation matters. The multicenter SLEEWE study (41) noted that while traditional sleep-stage percentages did not differ much between patients, those who passed a spontaneous breathing trial and were extubated reached higher levels of wakefulness (measured by a specific EEG metric, the odds ratio product during the preceding night). In contrast, patients who failed weaning showed abnormally low inter-hemispheric EEG coherence and pathological wakefulness and this EEG dyssynchrony strongly predicted weaning failure (AUC 0.91).

Broadly similar findings have been reported by other investigators. Shaikh et al. (42) studied prolonged mechanically ventilated patients (median duration of ventilation 38 days) in a weaning facility and found 11/44 (25%) had atypical sleep EEG patterns. Weaning failure was higher in the atypical sleep group (91%) than in the group with normal sleep (45%) (*p* = 0.013). Additionally, Marchasson et al. (43) pooled data from 131 conscious, non-sedated ICU patients, across three settings during the ICU stay: (1) Patients admitted for acute respiratory failure while breathing spontaneously (*n* = 34), (2) Patients undergoing mechanical ventilation with weaning difficulties (*n* = 45) and (3) Patients recently extubated (*n* = 52). Sleep was assessed by full PSG. The investigators found that deep sleep (N3) was relatively preserved in the spontaneously breathing group (Setting 1), but was markedly reduced in the patients under mechanical ventilation and after extubation (Settings 2 and 3). Atypical sleep patterns were significantly more frequent in the mechanically ventilated group compared to the spontaneously breathing group, while REM sleep was rare in all groups during their ICU stay. Patients with complete disappearance of REM sleep (about 50% of the cohort) had a higher rate of poor clinical outcomes (defined as need for intubation in the spontaneous group, prolonged weaning > 7 days under ventilator, or re-intubation after extubation) compared to those who had some REM sleep persistent.

Finally, Thille et al. (44) performed full PSG on the night immediately after extubation. They confirmed that overall sleep in these patients was profoundly poor (median total sleep of 2 to 3 h with 60% of patients exhibiting zero REM sleep). However, they observed no significant difference in sleep duration or architecture between patients who went on to develop post-extubation respiratory failure (or require reintubation) and those who did not. For example, reintubation occurred in 21% of patients with no REM vs. 5% with some REM, but this difference was not statistically significant. This negative finding suggests that by the time of extubation, sleep is disordered in most ICU patients. The study’s sample (52 patients) may have been underpowered to detect subtle effects, yet it’s possible that sleep prior to extubation (rather than after) is more influential on outcome.

### 3.2 Delirium

Short-term neurological outcomes, particularly ICU delirium, have been closely tied to sleep and circadian rhythm disturbances. An early study by Dessap et al. (45) showed that patients undergoing ventilator weaning who developed delirium had markedly altered circadian melatonin secretion patterns. Specifically, delirious patients produced lower nocturnal melatonin and higher cortisol, along with having almost no REM sleep compared to non-delirious controls. Low melatonin and minimal REM were significantly associated with delirium incidence, and the authors identified threshold values of REM ≤ 1% of sleep that optimally predicted delirium onset. These findings highlight a plausible pathophysiologic link; loss of normal circadian hormone variation and REM sleep may precipitate or reflect ICU delirium.

Several observational studies have attempted to relate subjective or objective sleep metrics to daily delirium risk, with mixed results. A post-hoc analysis examined 100 ICU patients from a randomized trial of low-dose nocturnal dexmedetomidine vs. placebo and found no significant association between patients’ self-reported sleep quality each morning and their delirium status either the previous day or the following day (46). In other words, a patient’s perception of a bad night’s sleep did not reliably predict delirium occurrence in this cohort. This well-controlled analysis (part of a larger RCT on delirium prevention) adds a critical perspective: subjective sleep measures may lack the sensitivity to characterize the relationship between sleep and delirium.

By contrast, Van der Hoeven et al. (47) assessed 323 ICU patients using validated sleep questionnaires and nursing observations to estimate day-to-night sleep ratios. Patients had higher day-to-night sleep ratios in the 3 days prior to delirium onset, suggesting that worse sleep quality was associated with delirium onset. Interestingly, this study also found that benzodiazepine use was common (24% of nights) and associated with shorter sleep duration and quality. This was a single-center retrospective study that was subject to biases in sleep and delirium assessments. However, its large sample strengthen the evidence that sleep disruption often precedes delirium.

Additional findings come from recent objective sleep analyses. Cardiopulmonary coupling used as a ECG-based sleep quality metric in 135 ICU patients showed that those who became delirious had significantly less stable sleep and more fragmented sleep than those without delirium (48). In fact, stable sleep time less than 0.65 h was a strong predictor of delirium, with an AUC of 0.89 and with 84% sensitivity. Multivariate regression indicated that greater stable sleep was protective against delirium, whereas factors like older age and higher apnea-hypopnea index (sleep-disordered breathing) increased delirium risk.

In a prospective case–control study, Sun et al. (49) compared 31 ICU patients who developed delirium with 31 matched controls without delirium. All participants underwent overnight PSG, actigraphy, and blood sampling for melatonin and cortisol to assess sleep architecture and circadian rhythmicity. Delirious patients showed severely disrupted sleep, with markedly reduced total sleep time, almost absent REM sleep, and greater sleep fragmentation. They also exhibited a blunted circadian rhythm, characterized by loss of day to night variation in melatonin and cortisol and flattened temperature and activity cycles. Overall, the study demonstrated that sleep and circadian rhythm disruption were strongly associated with ICU-acquired delirium and correlated with its severity and duration.

### 3.3 Brain recovery

EEG markers could help predict brain dysfunction and delirium. An interesting study investigated how noise sensitivity during sleep affects outcomes in non-sedated, mechanically ventilated ICU patientsv (50). Using full polysomnography combined with EEG markers of cortical reactivity to environmental noise, the authors quantified each patient’s noise-related arousal index. They found that patients who exhibited higher EEG reactivity to ambient noise had significantly longer weaning durations and more sleep fragmentation, despite similar sound pressure levels in the ICU. REM and slow-wave sleep were both markedly reduced in noise-sensitive patients. The authors suggested that individual sensitivity to noise could be a marker of brain function.

In a similar vein, investigators have recently studied the relationship between circadian rhythms and brain recovery after acute brain injury. In a recent study (51) performed in patients with disorders of consciousness, the investigators performed 24-h EEG monitoring and measured urinary melatonin and cortisol every two hours. Patients who displayed preserved circadian rhythmicity were significantly more likely to experience better neurological outcomes. The strength and regularity of these 24-h rhythms emerged as an independent prognostic marker. This larger prospective cohort supports the hypothesis that preserved or re-emerging circadian rhythms reflects a brain recovery, in line with an report of two comatose patients in whom circadian recovery paralleled cognitive improvement (52).

### 3.4 Inflammation

Sleep loss promotes a pro-inflammatory phenotype through activation of innate immune pathways and altered regulation of inflammatory gene expression. At the molecular level, clock genes directly regulate expression of inflammatory mediators including IL-6, TNF-*α*, and COX-2, and modulate innate immune cell function through circadian control of TLR9 trafficking and macrophage polarization (53). The mechanisms linking sleep/circadian disruption to inflammation in critical illness are likely bidirectional and multifactorial. In critically ill patients, systemic inflammation may itself disrupt clock gene expression, creating a vicious cycle wherein inflammation impairs circadian organization, which in turn exacerbates inflammatory responses.

Some studies have reported that critical illness frequently alters circadian regulated physiology, mainly metabolic and inflammation mechanisms based on clock gene regulation (54–57). For instance, Acuña-Fernández et al. (56) examined clock gene expression in septic shock patients. They found that septic ICU patients lost the normal daily oscillation of key clock genes (unlike non-septic ICU controls who maintained some rhythmicity). This circadian rhythm blunting in sepsis was correlated with increased inflammatory cytokines and oxidative stress markers. Interestingly, septic patients also showed elevated excretion of 6-sulfatoxymelatonin (aMT6s) correlated strongly with organ failure scores (SOFA) and procalcitonin. The authors suggested that a compensatory rise in melatonin translate an attempt to counteract inflammation/oxidative damage. These findings link circadian hormone disruption with the severity of critical illness: the worse the sepsis, the more circadian homeostasis is lost and the higher increase melatonin levels. The study was observational with a relatively small sample size (30 septic patients and 20 controls). The control ICU group showed that ICU conditions alone did not alter clock gene rhythms.

Additionally, Coiffard et al. (57) found that in severe trauma patients, all patients had disturbed circadian rhythms of cortisol, cytokines, leukocyte counts and clock genes, but those who subsequently developed sepsis displayed more pronounced rhythm disruption (higher cortisol mesor, delayed phase, altered leukocyte and cytokine rhythmicity), making early circadian dyssrhythmia a marker of later septic complications.

Finally, in COVID-19 ICU survivors, persistent circadian disruption correlates with ongoing inflammatory marker elevation, highlighting potential long-term consequences of ICU-acquired circadian misalignment (58).

### 3.5 Overall severity of illness

One of the first study by Varela et al. (59) demonstrated that loss of physiological temperature complexity is strongly associated with mortality in critically ill patients. Using continuous temperature recordings and nonlinear mathematical analysis, they showed that survivors maintained a more complex, variable, and structured temperature rhythm, whereas non-survivors displayed flattened, simplified, and highly predictable temperature curves. However, temperature should be interpreted cautiously as a circadian biomarker in ICU settings. Confounders include fever from infection, therapeutic hypothermia, environmental temperature variation, warming/cooling devices, antipyretic medications, and impaired autonomic thermoregulation. Thus, temperature complexity may reflect overall physiological variability rather than circadian rhythmicity per se, and should not be used as a sole marker of circadian function in the ICU.

Maas et al. (60) observed that ICU patients may enter a state of stress-induced behavioral quiescence characterized by profound inactivity and blunting of circadian activity rhythms, that was proportional to severity of illness. In 112 patients, they noted near-complete abolition of rest-activity cycles (far beyond what bedrest alone in healthy controls caused) and an absence of alignment between patients’ activity patterns and their melatonin secretion profiles. This state was not explained by delirium (both delirious and non-delirious patients showed behavioral quiescence) and may represent an adaptive, neurologically driven shutdown response in severe illness. While potentially protective, it also indicates that the circadian system is profoundly disturbed in the sickest patients.

More recently, Jiménez-Pastor et al. (61) showed that in ICU patients, melatonin levels and the expression of several clock genes (BMAL1, PER1, CLOCK, PER2, CRY2) were significantly altered according to illness severity and ICU length of stay, and that these molecular rhythms had non-linear associations with heart rate and blood pressure, linking circadian disruption to cardiovascular risk.

## 4 Long-term outcomes

### 4.1 Mortality

#### 4.1.1 Link to circadian alteration

Loss of the normal circadian pattern has been related to mortality. In a cohort of medical ICU patients, those who retained a robust 24-h melatonin rhythm (even if phase-shifted) had much better outcomes than those with arrhythmic or low-amplitude melatonin profiles. Specifically, having a detectable melatonin circadian rhythm was associated with five-fold higher odds of surviving to hospital discharge. Although this was a small study (37 patients), it positions melatonin rhythm as a potential biomarker of critical illness severity and survival. Patients with completely flat melatonin secretion tended to be the sickest and had the worst outcomes (62). This aligns with prior knowledge that severe inflammation (sepsis, trauma) can suppress normal hormone rhythms (54–56, 63).

#### 4.1.2 Link to sleep alteration

Beside circadian rhythms, alterations in EEG patterns have been associated with mortality. For instance, Boyko et al. (64) performed polysomnography on 52 ventilated ICU patients and found that the presence of atypical sleep EEG patterns was associated with an 11.6-fold higher odds of ICU mortality compared to patients with more normal sleep. Moreover, patients who lacked key stage N2 sleep features had significantly worse survival: absence of K-complexes carried an OR of 11 for ICU death, and absence of sleep spindles predicted higher in-hospital and 90-day mortality (OR 7.8 and 5.5, respectively). In survival analysis, loss of spindles correlated with a nearly fourfold increased hazard of 90-day mortality. This study is limited by its sample size and single-center design, yet it rigorously quantified EEG sleep and controlled for illness severity in analysis.

These results algin with those of Knauert et al. (65), who showed that ICU patients with delirium who lost stage N2 features had much higher in-hospital mortality. In a cohort of 93 delirious patients, those with no K-complexes or spindles on EEG had ORs of 18 and 6, respectively, for death before hospital discharge. Both studies, though observational, underscore a consistent point: the preservation of normal sleep EEG architecture (spindles, K-complexes) is an encouraging prognostic indicator, while diffuse atypical sleep leads to higher mortality.

A recent narrative review by Grigg-Damberger et al. (66) concluded that sleep spindles and K-complexes on ICU EEG are indeed favorable prognostic biomarkers. The review cites multiple studies where emergence of a more organized sleep pattern paralleled neurological recovery and better functional outcomes in brain-injured patients. It also notes evidence that sedative choice can influence EEG sleep quality. For example, patients who receive dexmedetomidine tend to have more spindles/K-complexes (a stage N2-like state) than those who receive propofol, which might contribute to better delirium outcomes in some trials. This highlights that improving sleep architecture in the ICU could translate to improved survival and long-term neurological benefits.

Beyond EEG-focused studies, broader analyses also connect sleep quality to mortality. Cai et al. (67) studied acute aortic dissection patients (a surgical ICU population) and found that those with poor pre-operative sleep quality had significantly higher in-hospital mortality than those with good sleep. Specifically, poor sleepers had over three times greater odds of dying in the hospital even after adjusting for clinical factors. They also had more post-operative complications like delirium and prolonged ventilation. While this study deals with a specific acute illness, it supports a generalizable point: sleep health prior to or during critical illness can influence survival. Patients entering critical care in a sleep-deprived or insomnia-prone state might be less resilient. This was a single-center study that relied on self-reported sleep questionnaires, so reporting bias is possible. Nevertheless, it suggests clinicians may want to screen for sleep problems as part of pre-operative risk stratification.

### 4.2 Post-intensive care syndrome (PICS)

#### 4.2.1 Prevalence of long-term sleep and circadian rhythm

Long term sleep quality, circadian rhythmicity, and their relationships with neurocognitive dysfunction in survivors of critical illness remains poorly studied. An emerging body of evidence links these domains, suggesting that sleep and circadian disruption during or after ICU might influence cognitive trajectories and mental health.

The prevalence of sleep and circadian disturbances in survivors of critical illness, as well as their pathogenesis, is uncertain. What is clear, however, is that such disturbances may be protracted. For instance, in the context of COVID-19 ICU survivors, Henríquez-Beltrán et al. (68) found that patients with the worst acute disease (long ICU stays and duration of mechanical ventilation) showed fragmented circadian rhythms up to 1 year later. This implies that circadian disruption might contribute to or indicate ongoing organ impairment in long-term survivors.

More recently, a prospective observational study recruiting adult survivors of severe acute respiratory failure who required invasive mechanical ventilation (69). Sleep quality was evaluated at two time points: (1) immediately before hospital discharge and (2) at six weeks post-discharge. Assessments included: the Richards-Campbell Sleep Questionnaire (RCSQ), the Insomnia Severity Index (ISI) and an overnight level 1 polysomnography. They found that survivors showed significant improvement in RCSQ. However, sleep disturbances persisted at six weeks follow-up, reflected mainly in the objective polysomnography metrics, which revealed altered sleep architecture, with reductions in total sleep time (427.5 ± 29.8), slow wave sleep (16.7 ± 2.7), lower sleep efficiency (68.5 ± 7.9) compared to normative data.

In a recent systematic review it was found that sleep disturbances are persistent for up to 12 months post-ICU, affecting a large proportion of patients (70). Subjective questionnaire studies revealed a 50–66.7% (within 1 month), 34–64.3% (1 to 3 months), 22–57% (3 to 6 months), and 10–61% (over 6 months). Only three studies reported subjective sleep assessment up to one-year after hospital discharge (71–73). Only four studies analyzed cohorts of over 20 patients, up to 6 months, with polysomnography (74–77). At 6 months, sleep architecture remained deeply altered with mean total sleep time less than 6 h, less than 15% of deep sleep by night and altered mean sleep efficiency (around 80%).

This review, though limited by heterogeneous studies, underscores that sleep disturbance after critical illness is not an isolated problem. The authors emphasize limitations in the evidence based due to methodological issues including the use of different sleep measurement tools and small sample sizes. Nonetheless, these data suggest that improving sleep may be critical for holistic recovery. Notably, the collection of symptoms that constitute post-intensive care syndrome (PICS) not only includes cognitive impairment, mental health, and physical alteration, but also sleep disturbances. Finally, fatigue, a common long-term complaint after critical illness (78) is interrelated with sleep and cognition, and may be linked to sleep disruption, although this has not been proven. More research is needed to understand how to specifically screen and address these long-term sleep and CR and their consequences on cognitive impairment, mental health and physical capacities.

#### 4.2.2 Cognitive outcomes

Sleep disruption negatively affects executive function, sustained attention, and memory consolidation (79), while increasing evidence implicates sleep disruption in the progression of neurodegenerative conditions like Alzheimer’s disease (80). Thus, recent studies have attempted to analyze the relationship between sleep and circadian disruption and long-term cognitive outcomes after critical illness (81–85). Ine one study (81) the investigators examined cognitive test scores in ICU survivors over time and related those to in-hospital sleep fragmentation measured via actigraphy. They found that greater sleep fragmentation in the ICU was associated with worse cognitive performance at hospital discharge, particularly in tests of short-term and delayed memory. This association, however, was no longer present at 6- and 12-month follow-up. This suggests that poor ICU sleep may delay or hinder early cognitive recovery, even if some patients eventually catch up cognitively after a year. Yet, the presence of an early cognitive deficit is itself prognostically relevant. Patients leaving the hospital with worse cognitive function may struggle more during rehabilitation and are at higher risk for hospital readmission or be unable to return to work. The methodology here (actigraphy for sleep, formal neuropsychological tests for cognition) was sound but limited by being a secondary analysis of a trial cohort and therefore not powered for this question. It nonetheless provides evidence linking sleep and CR in ICU to neurocognitive trajectories. In terms of cognitive function beyond 1 year, data are sparser and their relationship to sleep and circadian disruptions remains to be explored.

Of note, delirium confers a dose-dependent risk of persistent cognitive impairment (86), and is itself associated with sleep and circadian disruption, as discussed previously. Whether ICU-related sleep disruption accelerates neurodegenerative pathways or contributes to progression toward dementia is not yet established. Establishing a causal role for sleep and circadian disruption in the pathogenesis of long-term cognitive impairment is challenging given the potential for complex interactions between sleep, mood disorders, medication side effects, and other clinical phenomena that constitute the post-intensive care syndrome. These complexities emphasize the need for longitudinal neurocognitive follow-up studies integrating sleep biomarkers (87).

#### 4.2.3 Mental health

Anxiety, depression, and post-traumatic stress disorder have been linked to ICU sleep disruption as well. Jaiswal’s actigraphy study (88) while primarily about delirium, noted anecdotal trends that patients with more disrupted circadian activity in ICU tended to report worse anxiety scores later. Furthermore, a 12-month follow-up study demonstrated strong correlations between poor sleep quality and psychological distress in COVID-19 ICU survivors (68). At one year, patients with higher Pittsburgh Sleep Quality Index (PSQI) scores (indicating worse sleep) had significantly higher Hospital Anxiety and Depression Scale scores (rho: 0.5). It does not prove one causes the other, however, as both could be manifestations of underlying injury or ICU trauma, but it may be that they exacerbate each other. The study’s strength is its relatively large sample size (260 survivors) and the longitudinal use of both subjective (questionnaires) and objective (actigraphy) measures.

#### 4.2.4 Physical function

Long-term physical outcomes, such as muscle strength, mobility, and exercise capacity, are a major concern after critical illness. There is indirect evidence that early attention to sleep and wakefulness through sedation minimization and early mobilization yields better physical function at discharge, which may translate to improved long-term activity levels. Schweickert et al. (89) demonstrated that mechanically ventilated ICU patients who received daily interruption of sedation plus early physical/occupational therapy were far more likely to return to independent functional status by hospital discharge (59% in the intervention group vs. 35% in usual care, *p* < 0.05). They also had shorter durations of delirium and more ventilator-free days. This randomized trial’s methodology suggests that promoting daytime wakefulness and activity in ICU can improve functional outcomes. Because sleep and circadian rhythms were not directly measured, however, the mechanism of benefit cannot be ascertained, and more recent trials of early mobilization, albiet with different methodologies in different populations, have shown inconsistent results (90).

This early physical activity may improve sleep–wake cycling in a bidirectional way, patients in the intervention being awake more during day, which may improve night sleep. Still, research is lacking to understand how sleep and circadian rhythm can be addressed to improve post-ICU physical capacities.

## 5 Discussion

This review highlights the complex interactions between sleep disruption, circadian rhythm alterations, and clinical outcomes in critically ill patients. Across the studies included, sleep disturbances appear associated with different clinical consequences (Table 1). First, respiratory outcomes are frequently affected (38–41, 43–45). Sleep fragmentation and abnormal sleep architecture have been associated with impaired respiratory drive that delayed ventilator weaning, and prolonged mechanical ventilation. Additionally, several studies suggest a relationship between sleep disruption and delirium. Loss of circadian rhythmicity, reduced REM sleep, and fragmented sleep patterns appear to correlate with the development or persistence of ICU delirium (45–49). Sleep loss and circadian rhythm disturbances interact with systemic inflammation (53). Clinical studies suggest disruption of melatonin and cortisol rhythms may exacerbate inflammatory processes during critical illness (54–57). Overall, sleep disruption may influence global morbidity and mortality (62, 64–67). Emerging data suggest that sleep disturbances during ICU may contribute to long-term sequelae such as post-intensive care syndrome (PICS), including persistent cognitive impairments (69, 70, 81, 82, 84, 85).

**Table 1:** Summary of key findings on sleep and circadian dysrhythmias and outcomes in ICU patients.

Outcome domain	Timeframe	Key findings	Measurement modality
Delirium	Short-term	Delirious patients show reduced nocturnal melatonin, almost no REM sleep. Sleep fragmentation is associated to delirium risk.	Polysomnography/EEG Actigraphy Melatonin, cortisol
Cognitive function	Short-term	Sleep disruption during ICU stay is associated with worse cognitive performance at hospital discharge.	Actigraphy neuropsychological testing at discharge and follow-up
Mental health	Long-term	Sleep disruption in the ICU is associated with anxiety and psychological distress at 12 months.	Actigraphy Questionnaires (e.g., PSQI, HAD)
Inflammation	Short-term	Sleep loss and circadian misalignment is linked to elevated inflammatory cytokines and oxidative stress. Clock gene disruption accompanies worse inflammatory profiles. Melatonin metabolites (e.g., aMT6s) may rise with severity as a compensatory response.	Cytokines (IL-6, TNF-α) Clock gene expression Urinary melatonin metabolites (aMT6s)
Respiratory	Short-term	Poor sleep quality, atypical sleep EEG patterns, severe circadian disruption, and marked REM sleep reduction are associated with noninvasive ventilation failure, prolonged mechanical ventilation, difficult weaning, and poorer extubation-related outcomes.	Polysomnography Odds ratio product Weaning and extubation outcomes
Mortality	Short-term	Abnormal sleep EEG patterns (e.g., absence of K-complexes or spindles) are associated with higher ICU mortality	Polysomnography Mortality data
Mortality	Long-term	Circadian disruption markers (e.g., aMT6s) relate to poorer prognosis and survival	Melatonin rhythm profiling Mortality data

### 5.1 Limitations

A major limitation of the current literature relates to environmental and pharmacological confounding factors. The ICU environment itself profoundly disrupts sleep. Continuous noise, artificial lighting, frequent nursing interventions, and absence of natural day/night cues significantly alter circadian entrainment. In the settings of ICU, constant routine protocols cannot be applied to study the own rhythm of patient without confounders. Pharmacological exposure also represents a major source of bias. Sedatives and other medications commonly used in critical care significantly modify sleep architecture and EEG patterns (28, 29). These factors complicate interpretation of sleep measurements and limit the ability to distinguish disease-related sleep alterations from treatment effects or EEG abnormalities related to encephalopathy, seizures, or metabolic disturbances. Antibiotics, particularly fluoroquinolones and macrolides, can disrupt clock gene expression and alter inflammatory rhythms (91). Corticosteroids administered on fixed schedules may override endogenous cortisol rhythms. Catecholamine infusions, and opioid analgesics can similarly impact sleep and melatonin secretion (19). The cumulative impact of polypharmacy on ICU sleep and circadian biology remains poorly characterized but is likely substantial.

Additionally, patient populations differ considerably across studies. Cohorts may include medical ICU patients, postoperative patients, or neurocritical care populations, each with distinct pathophysiological mechanisms affecting sleep. In alert, non-sedated patients, polysomnography and melatonin profiling may be feasible and informative for guiding interventions such as timed light exposure. In deeply sedated or comatose patients, interpretation is limited, as sedatives profoundly modify sleep architecture and melatonin synthesis.

Sleep assessment methods vary widely between studies (questionnaires, polysomnography, actigraphy). Simpler tools such as actigraphy are highly susceptible to misclassification in immobile or sedated patients, while subjective questionnaires reflect perception rather than objective sleep and correlate inconsistently with objective measures (92). Melatonin profiling has emerged as a potential biomarker of circadian organization, yet its interpretation is complicated by altered light exposure, inflammation, organ dysfunction, and exogenous medication effects during critical illness (62).

An important limitation of the current evidence is that it primarily establishes associative rather than causal relationships. While sleep and circadian disruption correlate with adverse outcomes across multiple domains, the predominantly observational study designs, combined with substantial confounding from illness severity, sedation, and the ICU environment itself, preclude definitive conclusions regarding causality.

Finally, an additional layer of individual variability involves chronotype (morning vs. evening preference). No ICU studies have assessed chronotype or its interaction with ICU circadian disruption. It is plausible that pre-existing chronotype could modulate susceptibility to ICU-related misalignment or responsiveness to interventions such as timed light exposure. Future work should incorporate validated chronotype assessments to address this gap.

### 5.2 Clinical implications

Given the predominantly observational nature of the evidence and the multiple sources of confounding described above, interventions targeting sleep and circadian rhythms have not yet achieved strong guideline support. The distinction between biomarker identification and therapeutic intervention is critical: while disrupted sleep and circadian rhythms associate with worse outcomes, it does not necessarily follow that correcting these abnormalities will improve prognosis. These limitations explain why guideline recommendations remain elusive. Despite biological plausibility, most ICU sleep and circadian interventions have not reached universal guideline status. The society of critical care medicine focused update (93, 94) issued only a low-certainty recommendation for the use of melatonin. Current evidence suggests that melatonin supplementation may reduce the incidence of delirium in critically ill patients in pooled analyses of randomized trials. However, when delirium occurs, melatonin does not consistently reduce delirium duration, and there is no robust evidence for improvements in ICU length of stay, hospital stay, duration of mechanical ventilation, or mortality (95). Moreover, large pragmatic trials such as the DEMEL study (96) failed to demonstrate a clinically significant benefit in mechanically ventilated ICU patients, with uncertainty around optimal agent selection, dosing, and administration timing in relation to ICU-specific circadian phase shifts.

**Figure 1:**
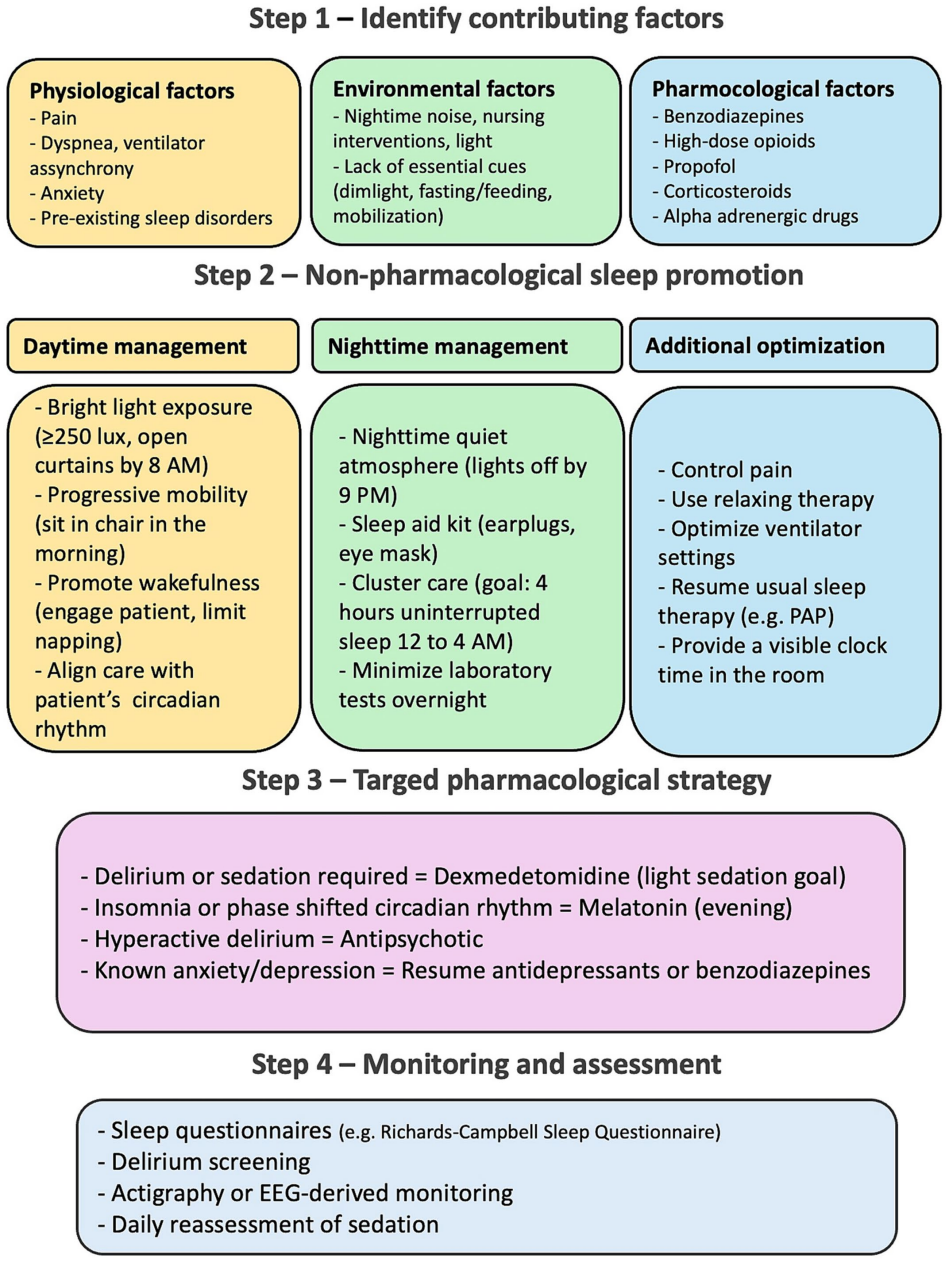
ICU patients with suspected sleep and circadian dysrhythmias.

Sleep and circadian rhythm restoration are likely to require multicomponent strategies (97), as suggested in Figure 1. A practical management algorithm should begin with daytime circadian reinforcement through appropriately timed light exposure, mobility promotion, and alignment of care activities with biological rhythms. Nighttime management should prioritize noise reduction, light minimization, clustering of care, and avoidance of unnecessary overnight interventions. Optimization of pain control, ventilator synchrony, and resumption of home sleep therapies should complement these measures. Pharmacologic therapy should be reserved for persistent disruption after non-pharmacologic optimization.

### 5.3 Future directions

Future research should therefore prioritize the development and validation of novel monitoring tools capable of reliably assessing sleep and circadian rhythms in critically ill patients. Simplified or miniaturized EEG-based devices use represent a promising avenue to capture sleep architecture and cortical function continuously and non-invasively (98–101). Advances in signal processing and automated analysis may further facilitate bedside implementation and interpretation tailored for ICU (31).

Pragmatic biomarker strategies using single or limited time-point measurements represent a promising avenue for ICU-compatible circadian assessment. Traditional circadian rhythm characterization requires serial sampling over 24 h, which is logistically challenging in critically ill patients. Recent advances suggest that molecular markers measured at one or two optimally selected time points may provide sufficient information to estimate circadian phase and amplitude (102). Methods such as a limited set of evening DLMO samples or a single-morning urinary 6-sulfatoxymelatonin measurement require validation in ICU populations. Development of ICU-specific circadian biomarker panels should be a research priority.

In parallel, large multicenter studies are needed to improve statistical power, enhance generalizability, and better characterize the relationship between sleep, circadian rhythms, and outcomes across different ICU populations. Collaborative research networks, particularly within intensive care medicine, offer an important opportunity to harmonize methodologies and data collection, enabling more robust conclusions. Long-term follow-up is another critical area for future investigation. Comprehensive assessments of cognitive, psychiatric, and physical outcomes beyond one year after ICU discharge are necessary to understand the persistence and clinical relevance of sleep and circadian disturbances acquired during critical illness (103).

## References

[1] Takahashi JS. Transcriptional architecture of the mammalian circadian clock. Nat Rev Genet. (2017) 18:164–79. doi: 10.1038/nrg.2016.150, 27990019 PMC5501165

[2] Allada R Bass J. Circadian mechanisms in medicine. N Engl J Med. (2021) 384:550–61. doi: 10.1056/NEJMra1802337, 33567194 PMC8108270

[3] Hastings MH Maywood ES Brancaccio M. Generation of circadian rhythms in the suprachiasmatic nucleus. Nat Rev Neurosci. (2018) 19:453–69. doi: 10.1038/s41583-018-0026-z, 29934559

[4] Welsh DK Takahashi JS Kay SA. Suprachiasmatic nucleus: cell autonomy and network properties. Annu Rev Physiol. (2010) 72:551–77. doi: 10.1146/annurev-physiol-021909-135919, 20148688 PMC3758475

[5] Saper CB Scammell TE Lu J. Hypothalamic regulation of sleep and circadian rhythms. Nature. (2005) 437:1257–63. doi: 10.1038/nature04284, 16251950

[6] Patke A Young MW Axelrod S. Molecular mechanisms and physiological importance of circadian rhythms. Nat Rev Mol Cell Biol. (2020) 21:67–84. doi: 10.1038/s41580-019-0179-2, 31768006

[7] Duffy JF Czeisler CA. Effect of light on human circadian physiology. Sleep Med Clin. (2009) 4:165–77. doi: 10.1016/j.jsmc.2009.01.004, 20161220 PMC2717723

[8] Czeisler CA Kronauer RE Allan JS Duffy JF Jewett ME Brown EN . Bright light induction of strong (type 0) resetting of the human circadian pacemaker. Science. (1989) 244:1328–33. doi: 10.1126/science.2734611, 2734611

[9] Evans JA Davidson AJ. Health consequences of circadian disruption in humans and animal models. Prog Mol Biol Transl Sci. (2013) 119:283–323. doi: 10.1016/B978-0-12-396971-2.00010-5, 23899601

[10] Musiek ES Holtzman DM. Mechanisms linking circadian clocks, sleep, and neurodegeneration. Science. (2016) 354:1004–8. doi: 10.1126/science.aah4968, 27885006 PMC5219881

[11] Kecklund G Axelsson J. Health consequences of shift work and insufficient sleep. BMJ. (2016):i5210. doi: 10.1136/bmj.i5210, 27803010

[12] Weingarten JA Collop NA. Air Travel. Chest. (2013) 144:1394–401. doi: 10.1378/chest.12-2963, 24081353

[13] Danielson SJ Rappaport CA Loher MK Gehlbach BK. Looking for light in the din: an examination of the circadian-disrupting properties of a medical intensive care unit. Intensive Crit Care Nurs. (2018) 46:57–63. doi: 10.1016/j.iccn.2017.12.006, 29605239 PMC6004325

[14] Telias I Wilcox ME. Sleep and circadian rhythm in critical illness. Crit Care. (2019) 23:82. doi: 10.1186/s13054-019-2366-0, 30850003 PMC6408803

[15] Cooper AB Thornley KS Young GB Slutsky AS Stewart TE Hanly PJ. Sleep in critically ill patients requiring mechanical ventilation. Chest. (2000) 117:809–18. doi: 10.1378/chest.117.3.809, 10713011

[16] Freedman NS Gazendam J Levan L Pack AI Schwab RJ. Abnormal sleep/wake cycles and the effect of environmental noise on sleep disruption in the intensive care unit. Am J Respir Crit Care Med. (2001) 163:451–7. doi: 10.1164/ajrccm.163.2.9912128, 11179121

[17] Kamdar BB Shah PA King LM Kho ME Zhou X Colantuoni E . Patient-nurse interrater reliability and agreement of the Richards-Campbell sleep questionnaire. Am J Crit Care. (2012) 21:261–9. doi: 10.4037/ajcc2012111, 22751369 PMC3667655

[18] Honarmand K Rafay H Le J Mohan S Rochwerg B Devlin JW . A systematic review of risk factors for sleep disruption in critically ill adults. Crit Care Med. (2020) 48:1066–74. doi: 10.1097/CCM.0000000000004405, 32433122

[19] Melone MA Gehlbach BK. "Characteristics of sleep in critically ill patients: part II: circadian rhythm disruption". In: Weinhouse GL Devlin JW, editors. Sleep in Critical Illness: Physiology, Assessment, and its Importance to ICU Care [Internet]. Cham: Springer International Publishing (2022). p. 15–36.

[20] Gazendam JAC Van Dongen HPA Grant DA Freedman NS Zwaveling JH Schwab RJ. Altered circadian rhythmicity in patients in the ICU. Chest. (2013) 144:483–9. doi: 10.1378/chest.12-2405, 23471224 PMC3734886

[21] Knauert MP Haspel JA Pisani MA. Sleep loss and circadian rhythm disruption in the intensive care unit. Clin Chest Med. (2015) 36:419–29. doi: 10.1016/j.ccm.2015.05.008, 26304279

[22] Kakar E Priester M Wessels P Slooter AJC Louter M van der Jagt M. Sleep assessment in critically ill adults: a systematic review and meta-analysis. J Crit Care. (2022) 71:154102. doi: 10.1016/j.jcrc.2022.154102, 35849874

[23] Drouot X Roche-Campo F Thille AW Cabello B Galia F Margarit L . A new classification for sleep analysis in critically ill patients. Sleep Med. (2012) 13:7–14. doi: 10.1016/j.sleep.2011.07.012, 22153778

[24] Drouot X. "Atypical sleep and pathologic wakefulness". In: Weinhouse GL Devlin JW, editors. Sleep in Critical Illness: Physiology, Assessment, and its Importance to ICU Care. Cham: Springer International Publishing (2022)

[25] Watson PL Pandharipande P Gehlbach BK Thompson JL Shintani AK Dittus BS . Atypical sleep in ventilated patients: empirical electroencephalography findings and the path toward revised ICU sleep scoring criteria. Crit Care Med. (2013) 41:1958–67. doi: 10.1097/CCM.0b013e31828a3f75, 23863228 PMC3875326

[26] Gehlbach BK Chapotot F Leproult R Whitmore H Poston J Pohlman M . Temporal disorganization of circadian rhythmicity and sleep-wake regulation in mechanically ventilated patients receiving continuous intravenous sedation. Sleep. (2012) 35:1105–14. doi: 10.5665/sleep.1998, 22851806 PMC3397814

[27] Zafar SF Subramaniam T Osman G Herlopian A Struck AF. Electrographic seizures and ictal-interictal continuum (IIC) patterns in critically ill patients. Epilepsy Behav. (2020) 106:107037. doi: 10.1016/j.yebeh.2020.107037, 32222672

[28] Beck F Gosseries O Weinhouse GL Bonhomme V. "Normal sleep compared to altered consciousness during sedation". In: Weinhouse GL Devlin JW, editors. Sleep in Critical Illness. Cham: Springer International Publishing (2022)

[29] Tung A Mendelson WB. Anesthesia and sleep. Sleep Med Rev. (2004) 8:213–25. doi: 10.1016/j.smrv.2004.01.003, 15144963

[30] Guldenmund P Vanhaudenhuyse A Sanders RD Sleigh J Bruno MA Demertzi A . Brain functional connectivity differentiates dexmedetomidine from propofol and natural sleep. Br J Anaesth. (2017) 119:674–84. doi: 10.1093/bja/aex257, 29121293

[31] Weinhouse GL Kimchi E Watson P Devlin JW. Sleep assessment in critically ill adults: established methods and emerging strategies. Crit Care Explor. (2022) 4:e0628. doi: 10.1097/CCE.0000000000000628, 35156048 PMC8824402

[32] Berry RB Brooks R Gamaldo C Harding SM Lloyd RM Quan SF . AASM scoring manual updates for 2017 (version 2.4). J Clin Sleep Med. (2017) 13:665–6. doi: 10.5664/jcsm.6576, 28416048 PMC5406946

[33] Frisk U Olsson J Nylén P Hahn RG. Low melatonin excretion during mechanical ventilation in the intensive care unit. Clin Sci. (2004) 107:47–53. doi: 10.1042/CS20030374, 14982491

[34] Gehlbach BK Patel SB Van Cauter E Pohlman AS Hall JB Zabner J. The effects of timed light exposure in critically ill patients: a randomized controlled pilot clinical trial. Am J Respir Crit Care Med. (2018) 198:275–8. doi: 10.1164/rccm.201801-0170LE, 29529381 PMC6058987

[35] Sertaridou EN Chouvarda IG Arvanitidis KI Filidou EK Kolios GC Pnevmatikos IN . Melatonin and cortisol exhibit different circadian rhythm profiles during septic shock depending on timing of onset: a prospective observational study. Ann Intensive Care. (2018) 8:118. doi: 10.1186/s13613-018-0462-y, 30515638 PMC6279676

[36] Thomas M Sing H Belenky G Holcomb H Mayberg H Dannals R . Neural basis of alertness and cognitive performance impairments during sleepiness. I. Effects of 24 h of sleep deprivation on waking human regional brain activity. J Sleep Res. (2000) 9:335–52. doi: 10.1046/j.1365-2869.2000.00225.x, 11123521

[37] Drummond SPA Brown GG Gillin JC Stricker JL Wong EC Buxton RB. Altered brain response to verbal learning following sleep deprivation. Nature. (2000) 403:655–7. doi: 10.1038/35001068, 10688201

[38] Roche Campo F Drouot X Thille AW Galia F Cabello B d’Ortho MP . Poor sleep quality is associated with late noninvasive ventilation failure in patients with acute hypercapnic respiratory failure. Crit Care Med. (2010) 38:477–85. doi: 10.1097/CCM.0b013e3181bc8243, 19789439

[39] Thille AW Reynaud F Marie D Barrau S Rousseau L Rault C . Impact of sleep alterations on weaning duration in mechanically ventilated patients: a prospective study. Eur Respir J. (2018) 51:1702465. doi: 10.1183/13993003.02465-2017, 29519925

[40] Rault C Sangaré A Diaz V Ragot S Frat JP Raux M . Impact of sleep deprivation on respiratory motor output and endurance. A physiological study. Am J Respir Crit Care Med. (2020) 201:976–83. doi: 10.1164/rccm.201904-0819OC, 31810378

[41] Dres M Younes M Rittayamai N Kendzerska T Telias I Grieco DL Sleep and Pathological Wakefulness at Time of Liberation from Mechanical Ventilation (SLEEWE): A Prospective Multicenter Physiological Study. Am J Respir Crit Care Med. (2019) 199:1106–15. 30818966 10.1164/rccm.201811-2119OC

[42] Shaikh H Ionita R Khan U Park Y Jubran A Tobin MJ . Effect of atypical sleep EEG patterns on weaning from prolonged mechanical ventilation. Chest. (2024) 165:1111–9. doi: 10.1016/j.chest.2024.01.005, 38211699 PMC11214907

[43] Marchasson L Rault C Le Pape S Arrivé F Coudroy R Frat JP . Impact of sleep disturbances on outcomes in intensive care units. Crit Care. (2024) 28:331. doi: 10.1186/s13054-024-05118-4, 39385194 PMC11466020

[44] Thille AW Barrau S Beuvon C Marie D Reynaud F Bardin J . Role of sleep on respiratory failure after extubation in the ICU. Ann Intensive Care. (2021) 11:71. doi: 10.1186/s13613-021-00863-z, 33963951 PMC8105690

[45] Dessap AM Roche-Campo F Launay JM Charles-Nelson A Katsahian S Brun-Buisson C . Delirium and circadian rhythm of melatonin during weaning from mechanical ventilation: an ancillary study of a weaning trial. Chest. (2015) 148:1231–41. doi: 10.1378/chest.15-0525, 26158245

[46] Duprey MS Devlin JW Skrobik Y. Is there an association between subjective sleep quality and daily delirium occurrence in critically ill adults? A post hoc analysis of a randomised controlled trial. BMJ Open Respir Res. (2020) 7:e000576. doi: 10.1136/bmjresp-2020-000576, 32847946 PMC7451265

[47] Van Der Hoeven AE Bijlenga D Van Der Hoeven E Schinkelshoek MS Hiemstra FW Kervezee L . Sleep in the intensive and intermediate care units: exploring related factors of delirium, benzodiazepine use and mortality. Intensive Crit Care Nurs. (2024) 81:103603. doi: 10.1016/j.iccn.2023.103603, 38171236

[48] Liu C Ji X Lu J Zhong L Hu J Wang Y . Assessment of sleep quality using cardiopulmonary coupling and its predictive value for delirium in ICU patients. Sleep Med. (2025) 126:222–7. doi: 10.1016/j.sleep.2024.12.020, 39705984

[49] Sun T Sun Y Huang X Liu J Yang J Zhang K . Sleep and circadian rhythm disturbances in intensive care unit (ICU)-acquired delirium: a case–control study. J Int Med Res. (2021) 49:030006052199050. doi: 10.1177/0300060521990502, 33730927 PMC7983249

[50] Rault CCS Frat JP Heraud Q Ragot S Coudroy R Melone MA . Noise-sensitivity during sleep in non-sedated mechanically ventilated patients is associated with weaning duration: a polysomnographic study. Sleep Breath. (2025) 29:19. doi: 10.1007/s11325-024-03207-w, 39607605

[51] Gobert F Corneyllie A Bastuji H Berthomier C Thevenet M Abernot J . Twenty-four-hour rhythmicities in disorders of consciousness are associated with a favourable outcome. Commun Biol. (2023) 6:1213. doi: 10.1038/s42003-023-05588-2, 38030756 PMC10687012

[52] Gobert F Luauté J Raverot V Cotton F Dailler F Claustrat B . Is circadian rhythmicity a prerequisite to coma recovery? Circadian recovery concomitant to cognitive improvement in two comatose patients. J Pineal Res. (2019) 66:e12555. doi: 10.1111/jpi.12555 30633817

[53] Irwin MR. Sleep and inflammation: partners in sickness and in health. Nat Rev Immunol. (2019) 19:702–15. doi: 10.1038/s41577-019-0190-z, 31289370

[54] Mundigler G Delle-Karth G Koreny M Zehetgruber M Steindl-Munda P Marktl W . Impaired circadian rhythm of melatonin secretion in sedated critically ill patients with severe sepsis*. Crit Care Med. (2002) 30:536–40. doi: 10.1097/00003246-200203000-00007, 11990911

[55] Li CX Liang DD Xie GH Cheng BL Chen QX Wu SJ . Altered melatonin secretion and circadian gene expression with increased proinflammatory cytokine expression in early-stage sepsis patients. Mol Med Rep. (2013) 7:1117–22. doi: 10.3892/mmr.2013.1331 23426900

[56] Acuña-Fernández C Marín JS Díaz-Casado ME Rusanova I Darias-Delbey B Pérez-Guillama L . Daily changes in the expression of clock genes in Sepsis and their relation with Sepsis outcome and urinary excretion of 6-Sulfatoximelatonin. Shock. (2020) 53:550–9. doi: 10.1097/SHK.0000000000001433, 31403491

[57] Coiffard B Diallo AB Culver A Mezouar S Hammad E Vigne C . Circadian rhythm disruption and Sepsis in severe trauma patients. Shock. (2019) 52:29–36. doi: 10.1097/SHK.0000000000001241, 30074979

[58] Livieratos A Lockley SW Tsiodras S. Post infectious fatigue and circadian rhythm disruption in long-COVID and other infections: a need for further research. EClinicalMedicine. (2025) 80:103073. doi: 10.1016/j.eclinm.2025.103073 39896874 PMC11787434

[59] Varela M Churruca J Gonzalez A Martin A Ode J Galdos P. Temperature curve complexity predicts survival in critically ill patients. Am J Respir Crit Care Med. (2006) 174:290–8. doi: 10.1164/rccm.200601-058OC, 16690981

[60] Maas MB Lizza BD Kim M Abbott SM Gendy M Reid KJ . Stress-induced behavioral quiescence and abnormal rest-activity rhythms during critical illness. Crit Care Med. (2020) 48:862–71. doi: 10.1097/CCM.0000000000004334, 32317592 PMC7242144

[61] Jiménez-Pastor JM Morales-Cané I Rodríguez-Cortés FJ López-Coleto L Valverde-León R Arévalo-Buitrago P . Interaction between clock genes, melatonin and cardiovascular outcomes from ICU patients. Intensive Care Med Exp. (2025) 13:19. doi: 10.1186/s40635-025-00730-2, 39961935 PMC11832861

[62] Melone MA Becker TC Wendt LH Ten Eyck P Patel SB Poston J . Disruption of the circadian rhythm of melatonin: a biomarker of critical illness severity. Sleep Med. (2023) 110:60–7. doi: 10.1016/j.sleep.2023.07.033, 37541132 PMC11386949

[63] Savaridas T Andrews PJD Harris B. Cortisol dynamics following acute severe brain injury. Intensive Care Med. (2004) 30:1479–83. doi: 10.1007/s00134-004-2306-5, 15138673

[64] Boyko Y Toft P Ørding H Lauridsen JT Nikolic M Jennum P. Atypical sleep in critically ill patients on mechanical ventilation is associated with increased mortality. Sleep Breath. (2019) 23:379–88. doi: 10.1007/s11325-018-1718-3 30215172

[65] Knauert MP Gilmore EJ Murphy TE Yaggi HK Van Ness PH Han L . Association between death and loss of stage N2 sleep features among critically ill patients with delirium. J Crit Care. (2018) 48:124–9. doi: 10.1016/j.jcrc.2018.08.028, 30179762 PMC6226351

[66] Grigg-Damberger MM Hussein O Kulik T. Sleep spindles and K-complexes are favorable prognostic biomarkers in critically ill patients. J Clin Neurophysiol. (2022) 39:372–82. doi: 10.1097/WNP.0000000000000830, 35239561

[67] Cai M Jiang F Lin L Peng Y Li S Chen L . Poor sleep quality is a risk factor for adverse clinical outcomes in patients with acute aortic dissection: a prospective cohort study. J Sleep Res. (2025) 34:e14411. doi: 10.1111/jsr.14411, 39568144 PMC12215227

[68] Henríquez-Beltrán M Vaca R Benítez ID González J Santisteve S Aguilà M . Sleep and circadian health of critical survivors: a 12-month follow-up study*. Crit Care Med. (2024) 52:1206–17. doi: 10.1097/CCM.0000000000006298, 38597721 PMC11239094

[69] Thulasi A Sharma VK Singh PK Ahuja A Arya G Chaudhry D. Quality of sleep in severe acute respiratory distress syndrome survivors requiring invasive mechanical ventilation: a prospective observational study. Sleep Breath. (2025) 29:137. doi: 10.1007/s11325-025-03304-4, 40131567

[70] Altman MT Knauert MP Pisani MA. Sleep disturbance after hospitalization and critical illness: a systematic review. Ann Am Thorac Soc. (2017) 14:1457–68. doi: 10.1513/AnnalsATS.201702-148SR, 28644698 PMC5711402

[71] Combes A Costa MA Trouillet JL Baudot J Mokhtari M Gibert C . Morbidity, mortality, and quality-of-life outcomes of patients requiring ≥14 days of mechanical ventilation. Crit Care Med. (2003) 31:1373–81. doi: 10.1097/01.CCM.0000065188.87029.C3, 12771605

[72] Orwelius L Nordlund A Nordlund P Edéll-Gustafsson U Sjöberg F. Prevalence of sleep disturbances and long-term reduced health-related quality of life after critical care: a prospective multicenter cohort study. Crit Care. (2008) 12:R97. doi: 10.1186/cc6973, 18673569 PMC2575585

[73] Parsons EC Hough CL Vitiello MV Zatzick D Davydow DS. Insomnia is associated with quality of life impairment in medical-surgical intensive care unit survivors. Heart Lung. (2015) 44:89–94. doi: 10.1016/j.hrtlng.2014.11.002, 25592203

[74] BaHammam A. Sleep quality of patients with acute myocardial infarction outside the CCU environment: a preliminary study. Med Sci Monit Int Med J Exp Clin Res. (2006) 12:CR168-172. 16572051

[75] Bahammam A Al-Mobeireek A Al-Nozha M Al-Tahan A Binsaeed A. Behaviour and time-course of sleep disordered breathing in patients with acute coronary syndromes: SDB and acute coronary syndromes. Int J Clin Pract. (2005) 59:874–80. doi: 10.1111/j.1742-1241.2005.00534.x, 16033605

[76] Schiza SE Simantirakis E Bouloukaki I Mermigkis C Kallergis EM Chrysostomakis S . Sleep disordered breathing in patients with acute coronary syndromes. J Clin Sleep Med. (2012) 8:21–6. doi: 10.5664/jcsm.1652, 22334805 PMC3266342

[77] Schiza SE Simantirakis E Bouloukaki I Mermigkis C Arfanakis D Chrysostomakis S . Sleep patterns in patients with acute coronary syndromes. Sleep Med. (2010) 11:149–53. doi: 10.1016/j.sleep.2009.07.016, 20083431

[78] Wintermann GB Rosendahl J Weidner K Strauß B Hinz A Petrowski K. Self-reported fatigue following intensive care of chronically critically ill patients: a prospective cohort study. J Intensive Care. (2018) 6:27. doi: 10.1186/s40560-018-0295-7, 29744108 PMC5930426

[79] Lowe CJ Safati A Hall PA. The neurocognitive consequences of sleep restriction: a meta-analytic review. Neurosci Biobehav Rev. (2017) 80:586–604. doi: 10.1016/j.neubiorev.2017.07.010, 28757454

[80] Gottesman RF Lutsey PL Benveniste H Brown DL Full KM Lee JM . Impact of sleep disorders and disturbed sleep on brain health: a scientific statement from the American Heart Association. Stroke. (2024) 55:e61–76. doi: 10.1161/STR.0000000000000453, 38235581

[81] Wilcox ME McAndrews MP Van J Jackson JC Pinto R Black SE . Sleep fragmentation and cognitive trajectories after critical illness. Chest. (2021) 159:366–81. doi: 10.1016/j.chest.2020.07.036, 32717265

[82] Daou M Telias I Younes M Brochard L Wilcox ME. Abnormal sleep, circadian rhythm disruption, and delirium in the ICU: are they related? Front Neurol. (2020) 11:549908. doi: 10.3389/fneur.2020.549908, 33071941 PMC7530631

[83] Wilcox ME Lim AS McAndrews MP Wennberg RA Pinto RL Black SE . A study protocol for an observational cohort investigating COGnitive outcomes and WELLness in survivors of critical illness: the COGWELL study. BMJ Open. (2017) 7:e015600. doi: 10.1136/bmjopen-2016-015600, 28710215 PMC5734403

[84] Daou M Lauzon C Bullen EC Telias I Fan E Wilcox ME. Long-term cognitive outcomes and sleep in adults after extracorporeal life support. Crit Care Explor. (2021) 3:e0390. doi: 10.1097/CCE.0000000000000390, 33834171 PMC8021370

[85] Taylor J Wilcox ME. Physical and cognitive impairment in acute respiratory failure. Crit Care Clin. (2024) 40:429–50. doi: 10.1016/j.ccc.2024.01.009, 38432704

[86] Fiest KM Soo A Hee Lee C Niven DJ Ely EW Doig CJ . Long-term outcomes in ICU patients with delirium: a population-based cohort study. Am J Respir Crit Care Med. (2021) 204:412–20. doi: 10.1164/rccm.202002-0320OC, 33823122 PMC8480248

[87] Rhodes A Wilson C Zelenkov D Adams K Poyant JO Han X . The psychiatric domain of post-intensive care syndrome: a review for the intensivist. J Intensive Care Med. (2025) 40:1223–39. doi: 10.1177/08850666241275582, 39169853

[88] Jaiswal SJ Bagsic SRS Takata E Kamdar BB Ancoli-Israel S Owens RL. Actigraphy-based sleep and activity measurements in intensive care unit patients randomized to ramelteon or placebo for delirium prevention. Sci Rep. (2023) 13:1450. doi: 10.1038/s41598-023-28095-0, 36702822 PMC9879948

[89] Schweickert WD Pohlman MC Pohlman AS Nigos C Pawlik AJ Esbrook CL . Early physical and occupational therapy in mechanically ventilated, critically ill patients: a randomised controlled trial. Lancet. (2009) 373:1874–82. doi: 10.1016/S0140-6736(09)60658-9, 19446324 PMC9906655

[90] The TEAM Study Investigators and the ANZICS Clinical Trials Group. Early active mobilization during mechanical ventilation in the ICU. N Engl J Med. (2022) 387:1747–58. doi: 10.1056/NEJMoa2209083, 36286256

[91] Livieratos A Grigoropoulos I Tsiodras S. Circadian considerations in antimicrobials. Eur J Clin Pharmacol. (2026) 82:73. doi: 10.1007/s00228-026-04002-0, 41649575

[92] Beecroft JM Ward M Younes M Crombach S Smith O Hanly PJ. Sleep monitoring in the intensive care unit: comparison of nurse assessment, actigraphy and polysomnography. Intensive Care Med. (2008) 34:2076–83. doi: 10.1007/s00134-008-1180-y, 18521566

[93] Lewis K Balas MC Stollings JL McNett M Girard TD Chanques G . A focused update to the clinical practice guidelines for the prevention and Management of Pain, anxiety, agitation/sedation, delirium, immobility, and sleep disruption in adult patients in the ICU. Crit Care Med. (2025) 53:e711–27. doi: 10.1097/CCM.0000000000006574, 39982143

[94] Hamilton DK Gary JC Scruth E Anderson HL Cadenhead CD Oczkowski SJ . Society of Critical Care Medicine 2024 guidelines on adult ICU design. Crit Care Med. (2025) 53:e690–700. doi: 10.1097/CCM.0000000000006572, 39982130

[95] Wu X Hu P Wu H Zhang K Ying J. Melatonin supplementation reduces delirium incidence in critically ill patients: a systematic review and meta-analysis. Front Pharmacol. (2026) 17:1728873. doi: 10.3389/fphar.2026.1728873, 41602948 PMC12832284

[96] Mekontso Dessap A Ricard JD Contou D Desnos C Decavèle M Sonneville R . Melatonin for prevention of delirium in patients receiving mechanical ventilation in the intensive care unit: a multiarm multistage adaptive randomized controlled clinical trial (DEMEL). Intensive Care Med. (2025) 51:1292–305. doi: 10.1007/s00134-025-08002-z, 40608082 PMC12283818

[97] Korwin AS Knauert MP. "Best practice for improving sleep in the ICU. Part I: non-pharmacologic". In: Weinhouse GL Devlin JW, editors. Sleep in Critical Illness. Cham: Springer International Publishing (2022)

[98] Peter-Derex L Berthomier C Taillard J Berthomier P Bouet R Mattout J . Automatic analysis of single-channel sleep EEG in a large spectrum of sleep disorders. J Clin Sleep Med. (2021) 17:393–402. doi: 10.5664/jcsm.8864, 33089777 PMC7927318

[99] Berthomier C Drouot X Herman-Stoïca M Berthomier P Prado J Bokar-Thire D . Automatic analysis of single-channel sleep EEG: validation in healthy individuals. Sleep. (2007) 30:1587–95. doi: 10.1093/sleep/30.11.1587, 18041491 PMC2082104

[100] Rault C Heraud Q Ragot S Frat JP Thille AW Drouot X. A real-time automated sleep scoring algorithm to detect refreshing sleep in conscious ventilated critically ill patients. Neurophysiol Clin. (2023) 53:102856. doi: 10.1016/j.neucli.2023.102856, 36966728

[101] De Gans CJ Burger P Van Den Ende ES Hermanides J Nanayakkara PWB Gemke RJBJ . Sleep assessment using EEG-based wearables – a systematic review. Sleep Med Rev. (2024) 76:101951. doi: 10.1016/j.smrv.2024.101951, 38754209

[102] Laing EE Möller-Levet CS Poh N Santhi N Archer SN Dijk DJ. Blood transcriptome based biomarkers for human circadian phase. eLife. (2017) 6:e20214. doi: 10.7554/eLife.20214, 28218891 PMC5318160

[103] Renner C Jeitziner MM Albert M Brinkmann S Diserens K Dzialowski I . Guideline on multimodal rehabilitation for patients with post-intensive care syndrome. Crit Care. (2023) 27:301. doi: 10.1186/s13054-023-04569-5, 37525219 PMC10392009

